# Supplementary material for: Crystal structures reveal catalytic and regulatory mechanisms of the dual-specificity ubiquitin/FAT10 E1 enzyme Uba6
Source: Nat Commun. 2022 Aug 19;13:4880. doi: 10.1038/s41467-022-32613-5 (PMC9391358; doi:10.1038/s41467-022-32613-5)
Supplement: Supplementary file 1 — Supplementary Information [file 41467_2022_32613_MOESM1_ESM.pdf]

Supplementary Information

Crystal Structures Reveal Catalytic and Regulatory Mechanisms of the Dual-Specificity Ubiquitin/FAT10 E1 Enzyme Uba6

Lingmin Yuan[1,8], Fei Gao[1,2,8], Zongyang Lv[1], Digant Nayak[1], Anindita Nayak[1], Priscila dos Santos Bury[1], Kristin E. Cano[1], Lijia Jia[1], Natalia Oleinik[3], Firdevs Cansu Atilgan[3], Besim Ogretmen[3], Katelyn M. Williams[4], Christopher Davies[5], Farid El Oualid[6], Elizabeth V. Wasmuth[1,7], & Shaun K. Olsen[1*]

[1]Department of Biochemistry & Structural Biology University of Texas Health Science Center at San Antonio, San Antonio, TX, 78229, USA

[2]Department of Research & Development, Beijing IPE Center for Clinical Laboratory CO. Beijing 100176, China

[3]Department of Biochemistry & Molecular Biology and Hollings Cancer Center, Medical University of South Carolina, Charleston, SC, 29425, USA

[4]Department of Pediatrics, Johns Hopkins University School of Medicine, Baltimore, MD, 21287, USA

[5]Department of Biochemistry & Molecular Biology, University of South Alabama, Mobile, AL, 36688, USA

[6]UbiQ Bio B.V., Science Park 408, 1098 XH, Amsterdam, The Netherlands

[7]Human Oncology and Pathogenesis Program, Memorial Sloan Kettering Cancer Center, New York, NY 10065, USA

[8]These authors contributed equally to this work

* Correspondence should be addressed to S.K.O. (olsens@uthscsa.edu)

Keywords: E1, ubiquitin, FAT10, IP6, inositol hexaskisphosphate, conformational change, adenylation, thioester

Supplementary Information Inventory:

Supplementary Table 1-2

Supplementary Figures 1-9

Supplementary Table 1 | Crystallographic Data and Refinement Statistics

	Uba6/ubiquitin-AMP/IP6 complex
PDB ID	7SOL
Source	APS 24 IDE
Wavelength (Å)	0.979
Resolution Limits (Å)	50.0-2.25 (2.29-2.25)
Space Group	*C*2
Unit Cell (Å) *a*, *b*, *c*	248.6, 101.3, 122.9
Unit Cell (°) α, β, γ	90.0, 118.0, 90.0
Number of observations	597982
Number of reflections	125930 (5797)
Completeness (%)	98.0 (91.1)
Mean I/σI	12.1 (1.5)
$CC_{1/2}$	0.996 (0.777)
R_{merge} [a]	0.083 (0.558)
R_{pim}	0.042 (0.283)
<u>Refinement Statistics</u>	
Resolution Limits (Å)	46.0-2.25 (2.31-2.25)
# of reflections (work/free)	123157/2000
Completeness (%)	98.0 (92.0)
Protein/solvent/ligand atoms	17035/847/82
R_{cryst} [b]	0.166 (0.215)
R_{free} (2000 reflections)	0.206 (0.275)
Bonds (Å)/ Angles (°)	0.004/0.718
B-factors: protein/solvent/ligand ($Å^2$)	54.5/45.3/48.4
Ramachandran plot statistics (%)	
favored	97.1
allowed	2.7
outliers	0.2
MolProbity Score	1.41- 99th percentile (N=11467, 2.25 Å ± 0.25 Å)

Parentheses indicate statistics for the high-resolution data bin for x-ray data.
a. $R_{merge} = \sum_{hkl} \sum_i |I(hkl)_i - \langle I(hkl)\rangle| / \sum_{hkl} \sum_i \langle I(hkl)_i\rangle$.
b. $R_{cryst} = \sum_{hkl} |F_o(hkl) - F_c(hkl)| / \sum_{hkl} |F_o(hkl)|$, where *Fo* and *Fc* are observed and calculated structure factors, respectively.

Supplementary Table 2 | All PCR primers used in these studies

Uba6_WT	
U6WT_F	GGTGGTCCATGGGAGTGGAAATCGATGATGCATTGTATAGTCGAC
U6WT_R	CCGGGCGGCCGCTTAATCAGTGTCATGACTGAAGTAGTATCTTACTGG
Uba6_C625A	
C625A_F	CCAGAAGAGGAAATACCATTTGCCACTCTAAAATCCTTTCCAGC
C625A_R	GCTGGAAAGGATTTTAGAGTGGCAAATGGTATTTCCTCTTCTGG
Uba6_E502A	
E502A_F	GATCCTGACTTGATAGCGAAATCCAACTTAAATAGA
E502A_R	TCTATTTAAGTTGGATTTCGCTATCAAGTCAGGATC
Uba6_D569A	
D569A_F	GTAATTATTACAGCATTAGCCAATGTGGAAGCCAGGAGAT
D569A_R	ATCTCCTGGCTTCCACATTGGCTAATGCTGTAATAATTAC
Uba6_K628D	
K628D_F	AAATACCATTTTGTACTCTAGATTCCTTTCCA
K628D_R	TGGAAAGGAATCTAGAGTACAAAATGGTATTT
Uba6_K714D	
K714D_F	GAAAAATATTTTAACCATGACGCTCTTCAGCTTCTTCACTGT
K714D_R	ACAGTGAAGAAGCTGAAGAGCGTCATGGTTAAAATATTTTTC
Uba6_ K644E/S648L/L702W/K706H/K709T/Y710Q- InsP6 binding site to Uba1	
RD1_F	CACCATACAGTGGGCAAGAGATGAATTTGAAAGTTTGTTTTCCCACAAACCTTCA
RD1_R	TGAAGGTTTGTGGGAAAACAAACTTTCAAATTCATCTCTTGCCCACTGTATGGTG
RD2_F	GAAATTGGTCCCAGTGTGTAGAATGGGCAAGATTACACTTTGAAAAATATTTTAACCA
RD2_R	TGGTTAAAATATTTTTCAAAGTGTAATCTTGCCCATTCTACACACTGGGACCAATTTC
RD3_F	GAATGGGCAAGATTACACTTTGAAACCCAGTTTAACCATAAGGCTCT
RD3_R	AGAGCCTTATGGTTAAACTGGGTTTCAAAGTGTAATCTTGCCCATTC
Ubiquitin_Cys0 mutant for CF488A fluorescent labeling	
UBC0_F	GTATTTTCAGGGCGCCATGGCTTGCATGCAGATCTTCGTGAAGA
UBC0_R	TCTTCACGAAGATCTGCATGCAAGCCATGGCGCCCTGAAAATAC
FAT10_CYS0 MUTANT FOR CF488A FLUORESCENT LABELING	
F10C0_F	CCAGGGGCCCCTGGGATCCTGCGCTCCTAATGCCTCTACCCT
F10C0_R	AGGGTAGAGGCATTAGGAGCGCAGGATCCCAGGGGCCCCTGG
FAT10_C7T/C9T/C134L/C160S/C162S	
Purchased from Gene Universal	

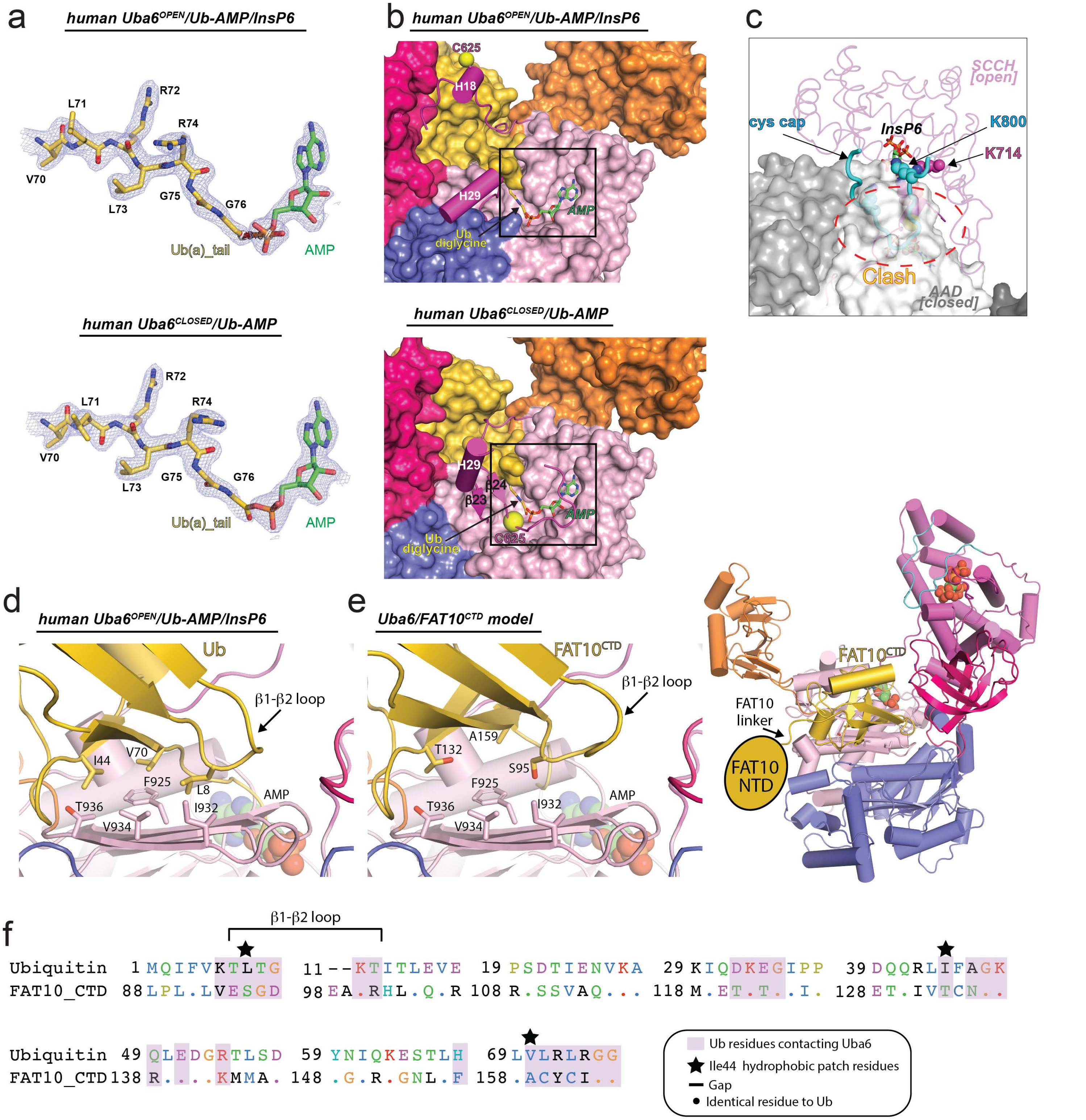

Supplementary Fig.1. Comparison of hUba6/Ub(a) complexes in open and closed states.

a, 2Fo-Fc electron density maps contoured at 1.5σ for Ub(a)-AMP in hUba6 open conformation and closed conformation. The electron density map is shown as slate mesh. Ubiquitin-AMP is shown as sticks. **b**, Comparison of Uba6 in open and closed conformations. Uba6 is shown as a surface representation, and the elements that under conformational changes in the Cys domain are shown as cartoon. **c**, SCCH domain from Uba6 [open] is superimposed onto the SCCH domain of Uba6 [closed], and the cys cap clashes with AAD from Uba6 [closed]. **d**,Cartoon representation of the Uba6/Ub interface. Residues from the Ile44 hyrophobic patch of Ub and interacting residues from Uba6 are shown as sticks. The β1-β2 loop of Ub is highlighted. **e**,Cartoon representation of a Uba6/FAT10 CTD complex. FAT10 CTD (PDB: 6GF2) was superposed onto Ub from the Uba6/Ub structure. Residues corresponding to the Ile44 hyrophobic patch of Ub and interacting residues from Uba6 are shown as sticks. The β1-β2 loop of Ub is highlighted. **f**, Sequnce alignment of Ub and FAT10 CTD. Ub residues that contact Uba6 in the Uba6/Ub structure are highlighted purple. Residues from the Ile44 hydrophobic patch are indicated with a black star. Dashes represent gaps and periods represent identical residues to Ub.

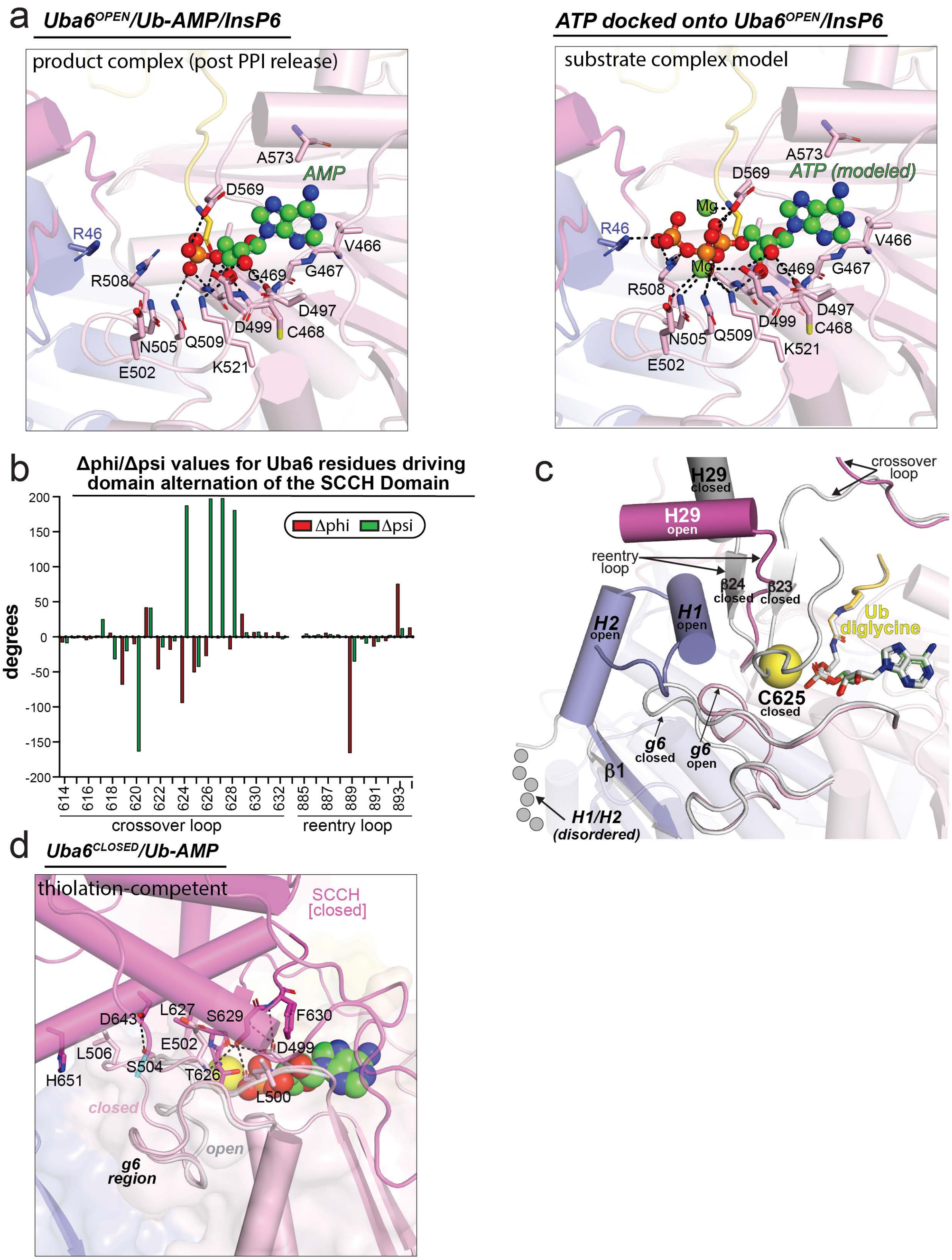

Supplementary Fig. 2. Active site remodeling and domain alternation in Uba6.

a, Overview of contacts between AMP and Uba6 in our Uba6^{OPEN}/Ub-AMP/InsP6 structure, which represents the product complex of adenylation, after pyrophoshpate (PPI) has been released from the active site (*Left*). A model of the substrate complex of adenylation, e.g. Uba6^{OPEN}/Ub/MgATP, was created by docking MgATP from PDB:4II2 onto our Uba6^{OPEN}/Ub-AMP/InsP6 structure (*Right*). Side chains of Uba6 residues which interact with nucleotide are shown as sticks. AMP, ATP, and Mg are shown as spheres. **b**, Variation of phi and psi angles of residues in crossoverand reentry loops between Uba6 open and closed conformations. **c**, Superposition of crossover and re-entry loops for Uba6 adenylate and thioester competent states. The Uba6 thioester competent state is colored gray. **d**, The interaction network between g6 region and SCCH domain in Uba6 closed conformation.

Source data are provided as a Source Data file.

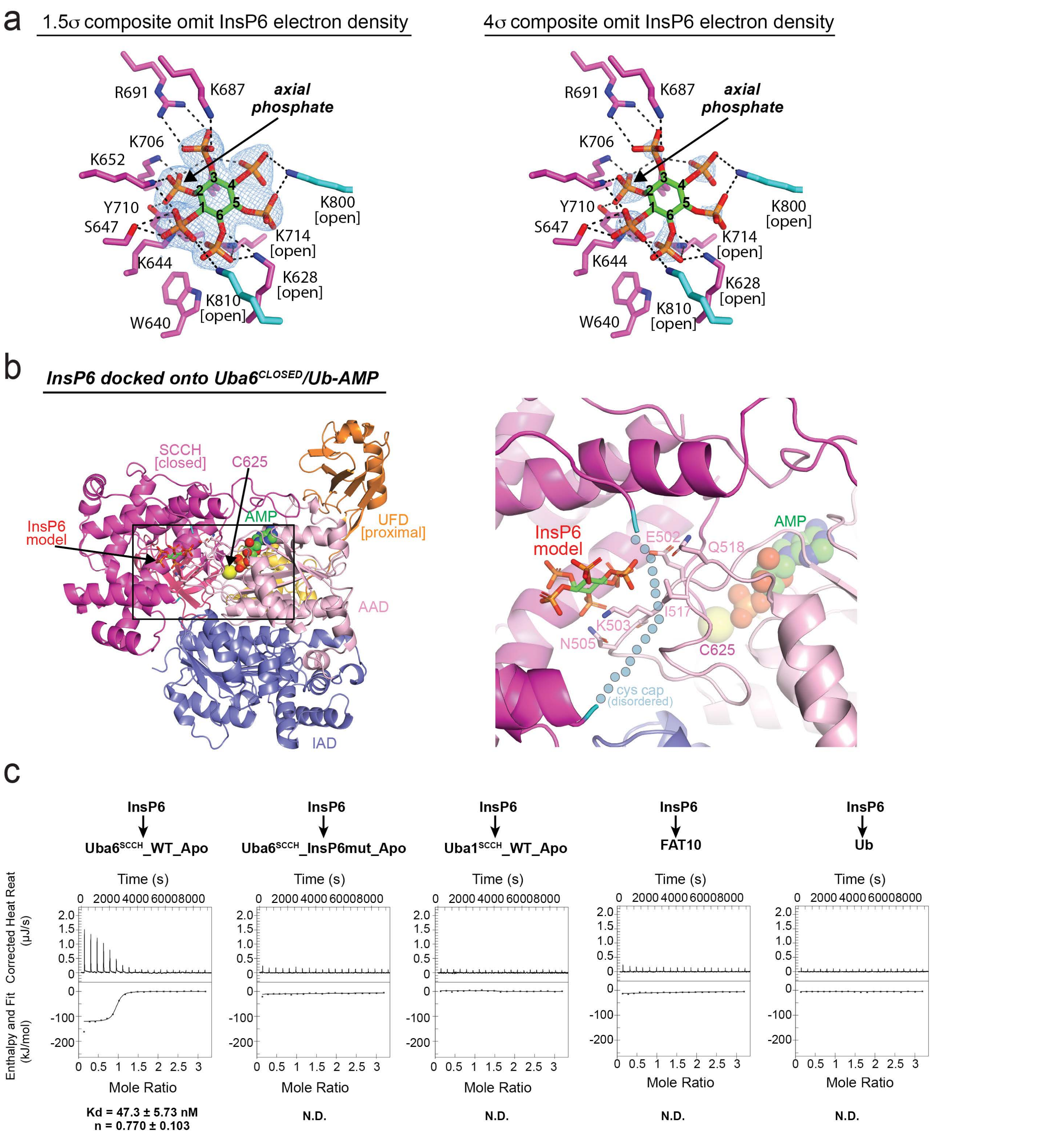

Supplementary Fig. 3. InsP6 specifically binds in the highly basic pocket of Uba6 SCCH domain.
a, Interaction between Uba6 and InsP6. The side chains of residues involved in direct contact with InsP6 are shown as sticks. InsP6 is shown as sticks with composite omit electron density maps shown as mesh. Sigma level at 1.5 on the left and 4 on the right. **b**, Model of a Uba6^{CLOSED}/Ub-AMP/Insp6 complex. InsP6 was docked onto the SCCH domain of the Uba6^{CLOSED}/Ub-AMP structure. The side chains of residues within 5 Å of InsP6 are shown as sticks. InsP6 is shown as spheres. The disordered Cys Cap is shown as semitransparent spheres. **c**, Affinity of InsP6 with SCCH domain fragments of Uba6_WT_Apo, Uba6_InsP6_Apo and Uba1_WT_Apo, ubiquitin and FAT10 tested by ITC. Throughout the figure, 'Apo' labels refers to *E.coli* derived material.

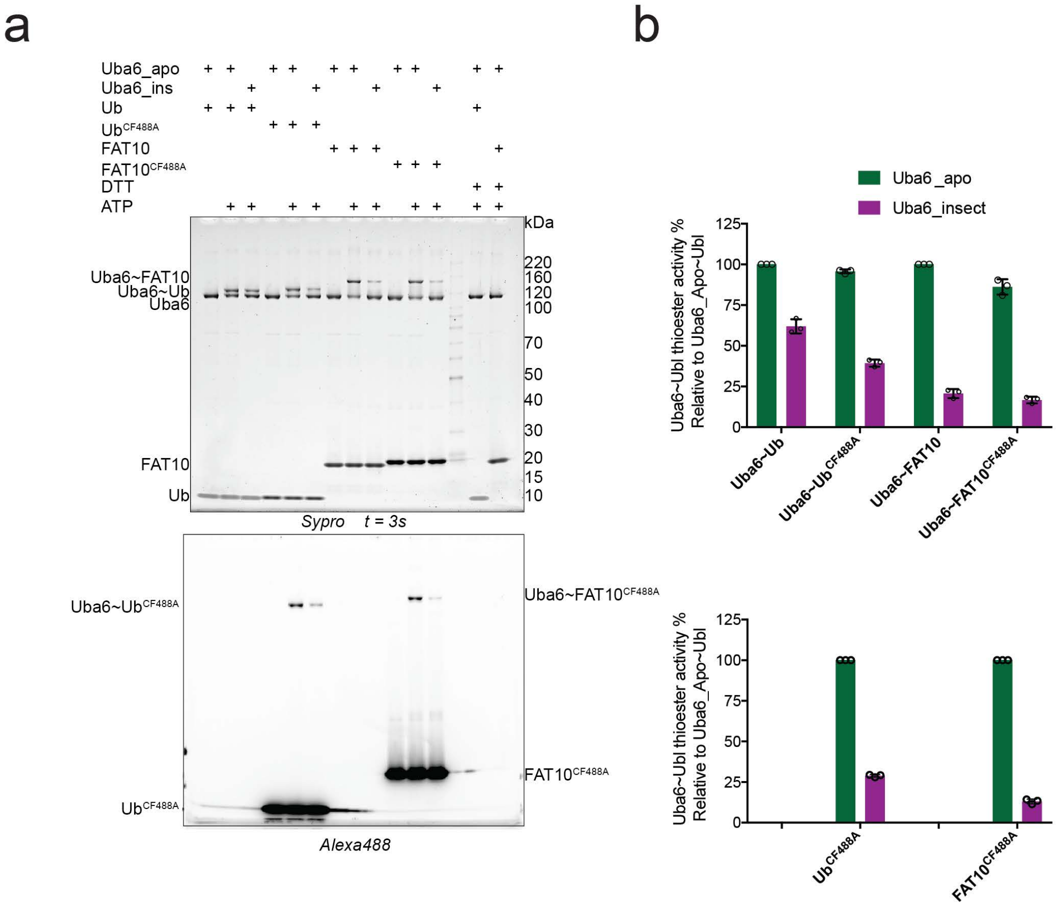

Supplementary Fig. 4. Uba6~Ub and Uba6~FAT10 thioester formation activities.

a, Activity of the indicated Uba6 variants in E1~Ubl thioester formation assays detected using unlabeled versus CF488A labeled Ubl. The gels presented are representative results from the n= 3 technical replicates independently repeated with similar results. Gels were first imaged imaged on a ChemiDoc MP (BioRad) using the Alex488 fluorescent channel (bottom). Gels were subsequently stained with Sypro Ruby (top). Source data are provided as a Source Data file. **b**, Data from panel **a** are presented in graph format as mean values +/- SEM (n= 3 technical replicates) with individual replicates shown as gray circles. Source data are provided as a Source Data file. Throughout the figure, Uba6_apo refers to *E. coli*-derived material and Uba6_ins refers to insect cell-derived material.

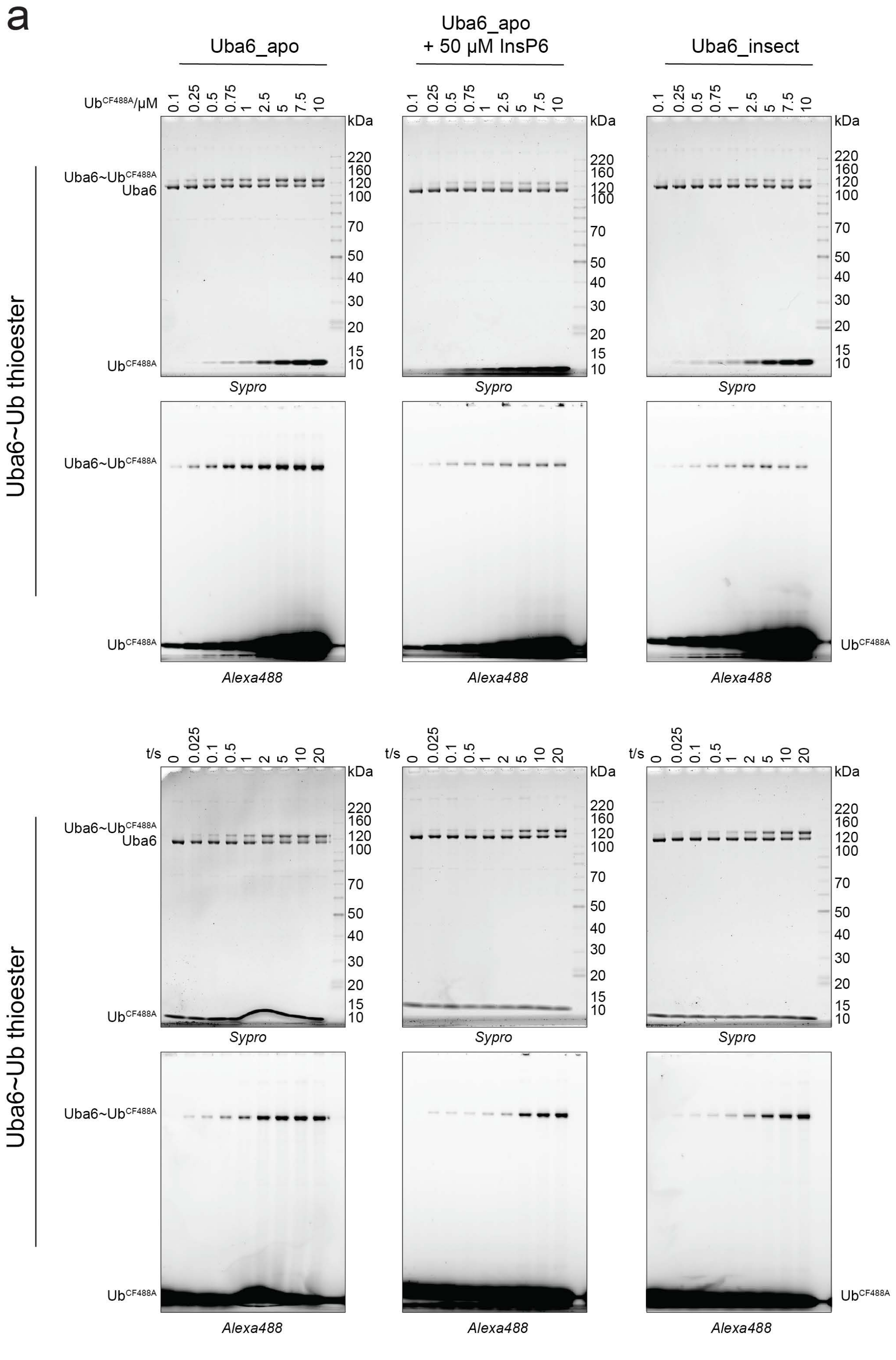

Supplementary Fig. 5. InsP6 effects on the kinetics of Uba6~Ub thioester formation activity

a, Raw data for kinetic studies of the indicated Uba6 variants in Uba6~Ub thioester formation assays at the indicated concentrations and time points. Kinetic parameters were calculated as described in the Methods and presented in Figure 4a and Table 1. The gels presented are representative results from the n= 3 technical replicates independently repeated with similar results. Gels were first imaged imaged on a ChemiDoc MP (BioRad) using the Alex488 fluorescent channel (bottom). Gels were subsequently stained with Sypro Ruby (top). Source data are provided as a Source Data file. Throughout the figure, Uba6_apo refers to *E. coli*-derived material and Uba6_insect refers to insect cell-derived material.

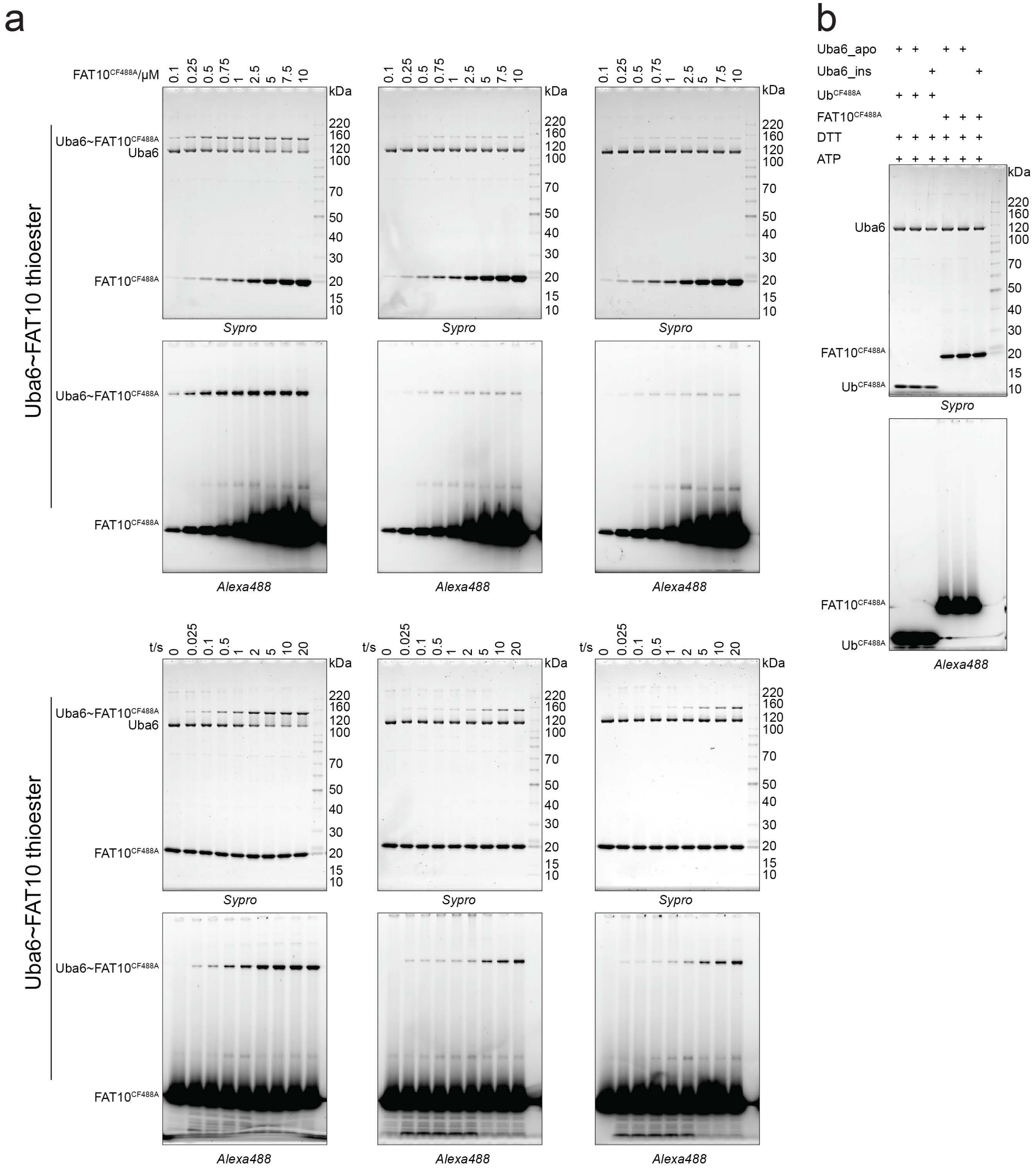

Supplementary Fig. 6. InsP6 effects on the kinetics of Uba6~FAT10 thioester formation activity

a, Raw data for kinetic studies of the indicated Uba6 variants in Uba6~FAT10 thioester formation assays at the indicated concentrations and time points. Kinetic parameters were calculated as described in the Methods and presented in Figure 4b and Table 1 . The gels presented are representative results from the n= 3 technical replicates independently repeated with similar results. Gels were first imaged imaged on a ChemiDoc MP (BioRad) using the Alex488 fluorescent channel (bottom). Gels were subsequently stained with Sypro Ruby (top). Source data are provided as a Source Data file. **b**, Control Uba6~Ub and Uba6~FAT10 thioester formation assays demonstrating the DTT sensitivity of the products which is consistent with thioester formation. Gels were first imaged imaged on a ChemiDoc MP (BioRad) using the Alex488 fluorescent channel (bottom). Gels were subsequently stained with Sypro Ruby (top). Throughout the figure, Uba6_apo refers to *E. coli*-derived material and Uba6_ins refers to insect cell-derived material.

a

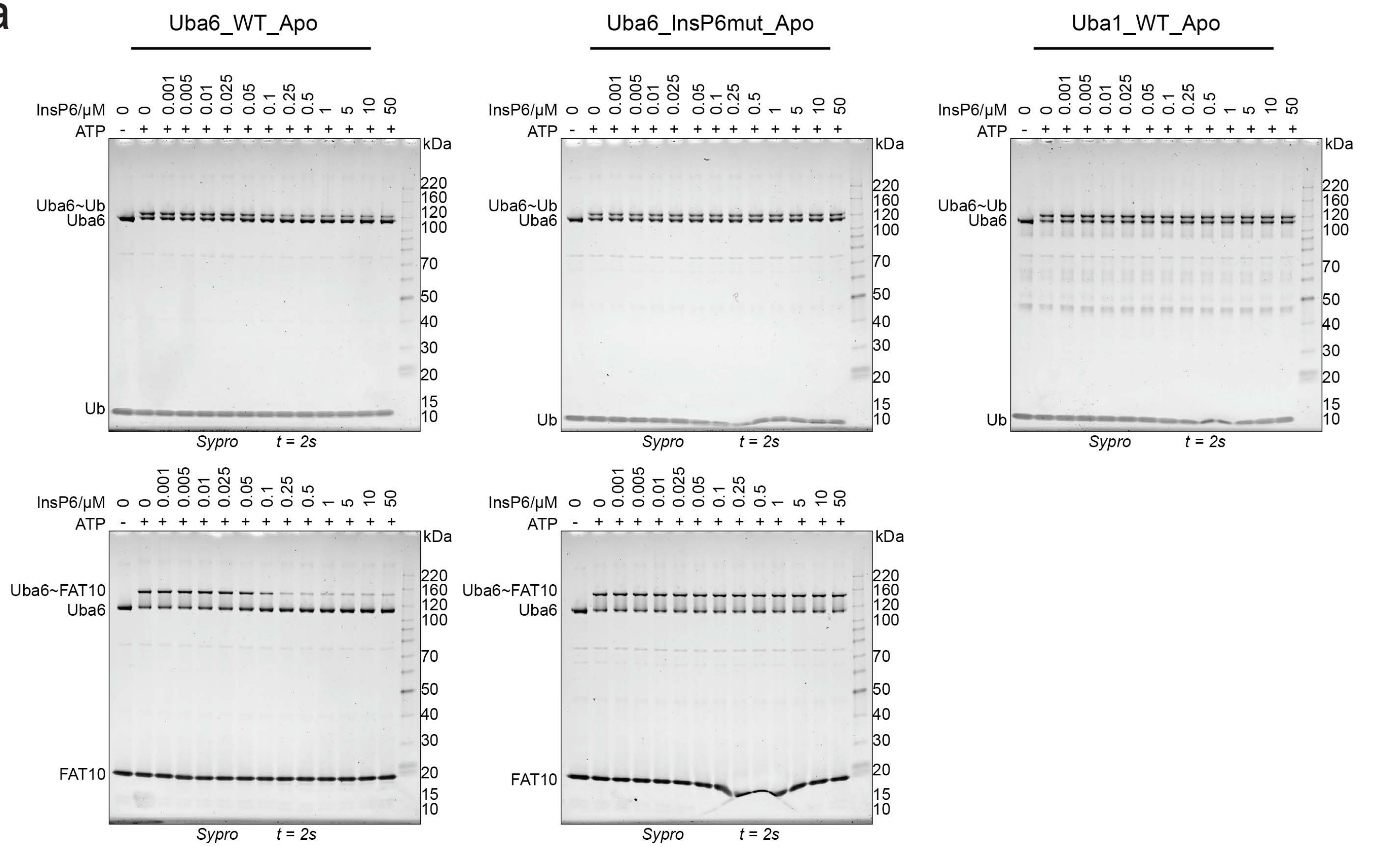

b

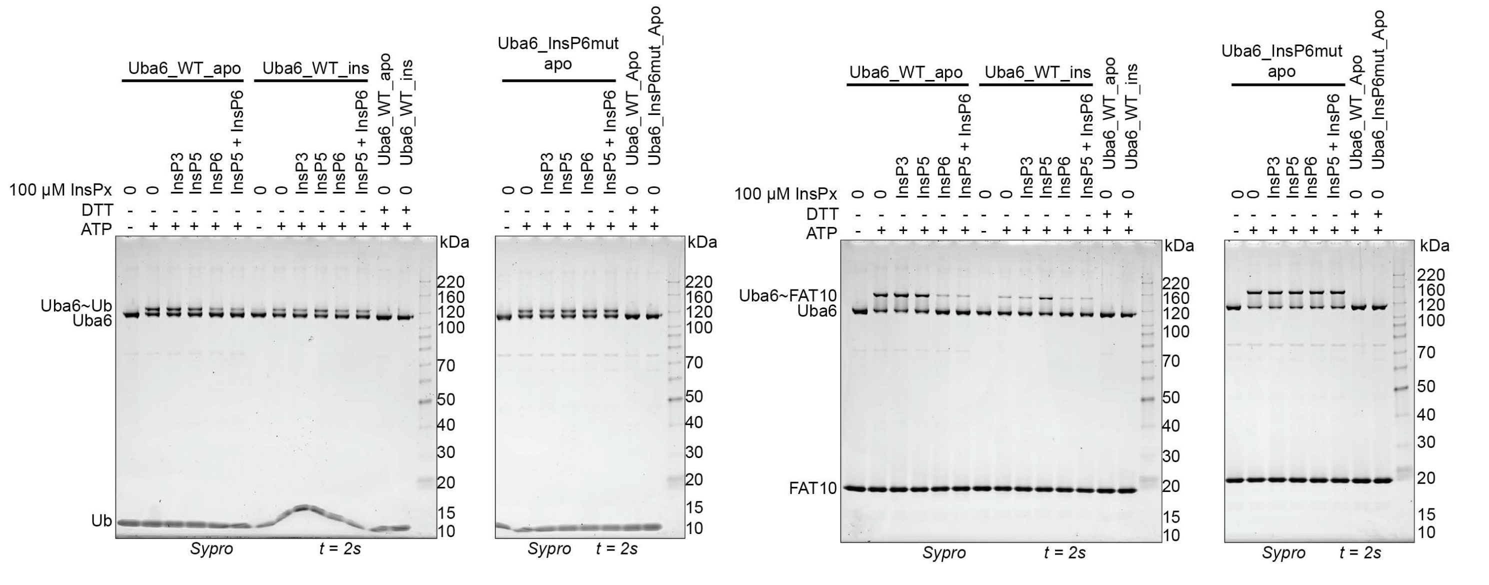

Supplementary Fig. 7. Effects of inositol phosphates on Uba6 E1~Ubl thioester formation activity.

a, Raw data for dose response of InsP6 on E1~Ubl thioester formation presented in Figure 4c. The gels presented are representative results from the n= 3 technical replicates independently repeated with similar results. Source data are provided as a Source Data file. **b**, Raw data for evaluation of the effectsof InsPx (3, 5 or 6) on E1~Ubl thioester formation presented in Figure 4d. The gels presented are representative results from the n= 3 technical replicates independently repeated with similar results. Source data are provided as a Source Data file. In all panels, Uba6_Apo refers to *E. coli*-dervived Uba6 and Uba6_ins refers to insect cell-derived material.

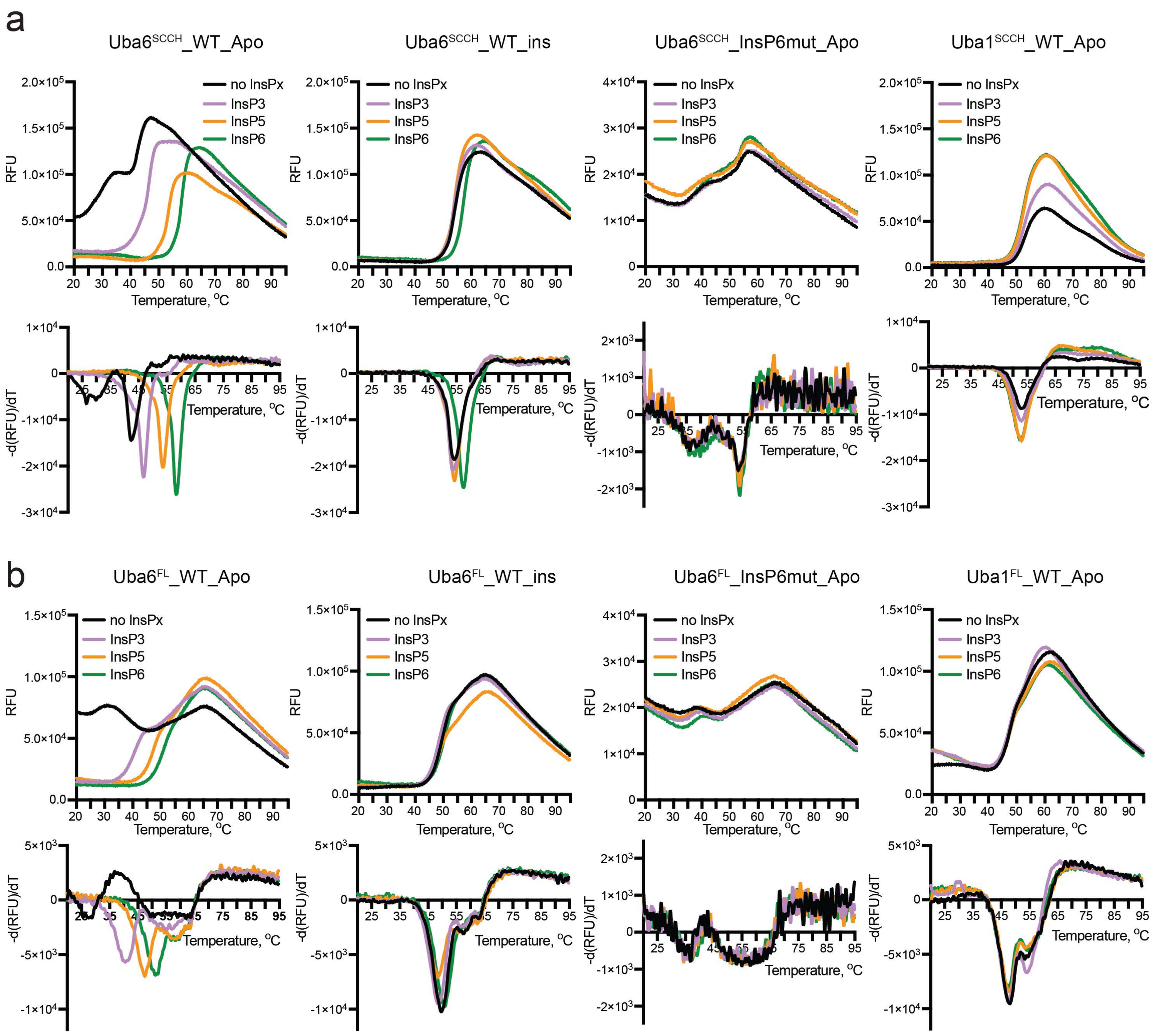

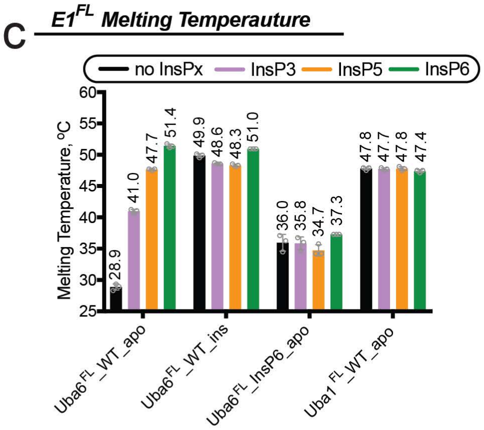

Supplementary Fig. 8. Effects of InsP6 on hUba6 stability

a, **b**, Raw data for determination of melting temperature of SCCH domain (panel **a**) and FL Uba6 (panel **b**) via Thermal Stability Assay. The data presented are representative results from the n= 3 technical replicates independently repeated with similar results. Source data are provided as a Source Data file. **c**, Data from panel **b** are presented in graphs as mean values +/- SEM (n= 3 technical replicates) with individual replicates shown as gray circles. Source data are provided as a Source Data file. Throughout the figure, Uba6_apo refers to E. coli-derived material and Uba6_ins refers to insect cell-derived material.

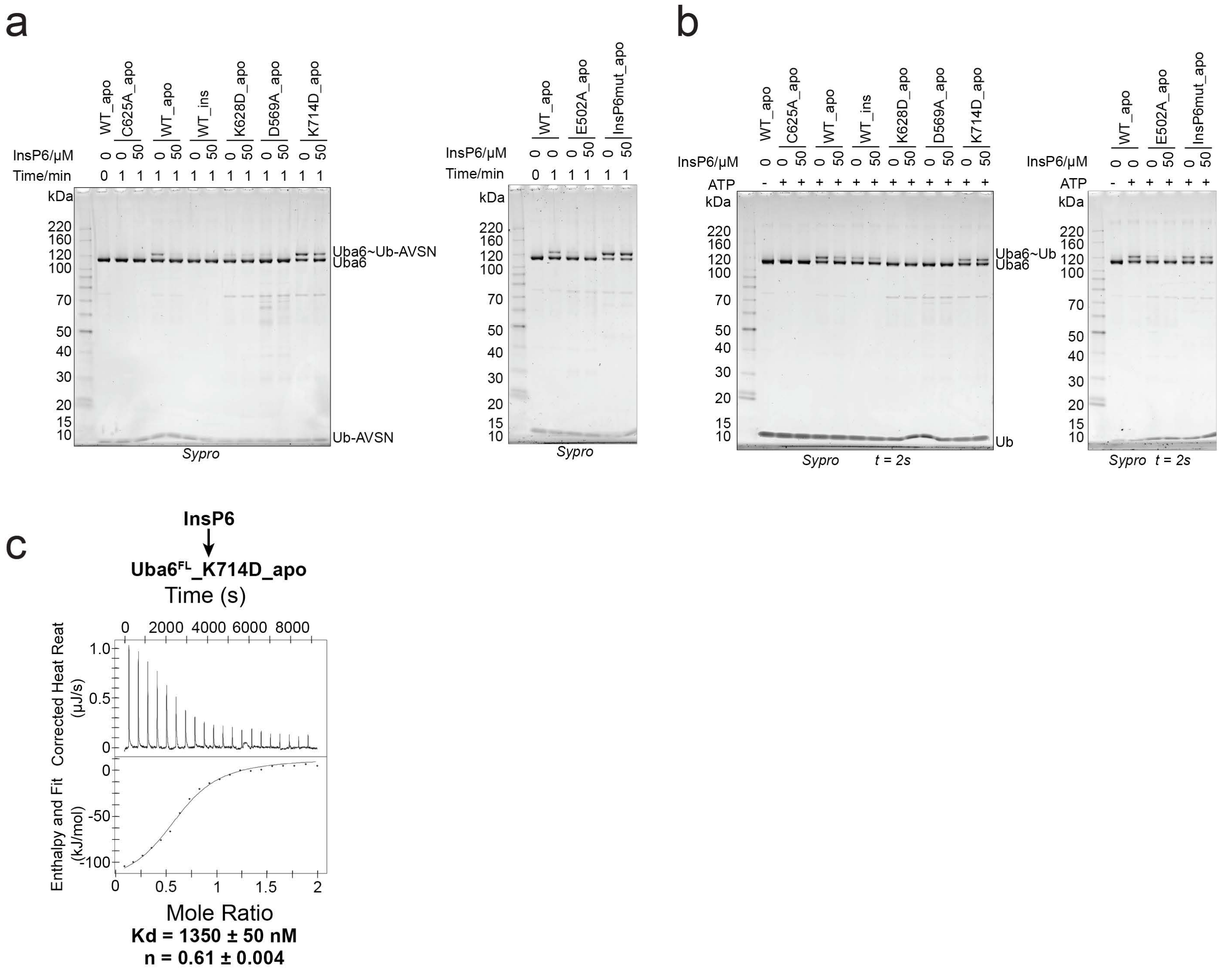

Supplementary Fig. 9. Effects of InsP6 on E1/Ub-AVSN crosslinking and Uba6 E1~Ubl thioester formation.

a, Raw data for E1/Ub-AVSN crosslink activity of Uba6 WT and mutants in the presence and absence of InsP6. This data is presented in Figure 5d and the gels presented are representative results from the n= 3 technical replicates independently repeated with similar results. Source data are provided as a Source Data file. **b**, Raw data for E1~Ub thoester formation activity of Uba6 WT and mutants in the presence and absence of InsP6. This data is presented in Figure 5e and the gels presented are representative results from the n= 3 technical replicates independently repeated with similar results. Source data are provided as a Source Data file. **c**, Isothermal titration calorimetry data for interaction between the K714D Uba6 mutant and InsP6. Experiments were performed in triplicate with similar results and upper panels show raw power data and lower panels show fits of the data to standard binding equations using NanoAnalyze software (TA instruments). 'Apo' labels refer to *E. coli*-derived material and 'ins' label refers to insect cell-derived material.
