# Supplementary material for: Crystal structures reveal catalytic and regulatory mechanisms of the dual-specificity ubiquitin/FAT10 E1 enzyme Uba6
Source: Nat Commun. 2022 Aug 19;13:4880. doi: 10.1038/s41467-022-32613-5 (PMC9391358; doi:10.1038/s41467-022-32613-5)
Supplement: Supplementary file 3 — Reporting Summary [file 41467_2022_32613_MOESM3_ESM.pdf]

nature portfolio

Corresponding author(s): Shaun K. Olsen

Last updated by author(s): Aug 1, 2022

Reporting Summary

Nature Portfolio wishes to improve the reproducibility of the work that we publish. This form provides structure for consistency and transparency in reporting. For further information on Nature Portfolio policies, see our Editorial Policies and the Editorial Policy Checklist.

Statistics

For all statistical analyses, confirm that the following items are present in the figure legend, table legend, main text, or Methods section.

n/a	Confirmed	
☐	☒	The exact sample size (n) for each experimental group/condition, given as a discrete number and unit of measurement
☐	☒	A statement on whether measurements were taken from distinct samples or whether the same sample was measured repeatedly
☒	☐	The statistical test(s) used AND whether they are one- or two-sided<br>*Only common tests should be described solely by name; describe more complex techniques in the Methods section.*
☒	☐	A description of all covariates tested
☒	☐	A description of any assumptions or corrections, such as tests of normality and adjustment for multiple comparisons
☐	☒	A full description of the statistical parameters including central tendency (e.g. means) or other basic estimates (e.g. regression coefficient) AND variation (e.g. standard deviation) or associated estimates of uncertainty (e.g. confidence intervals)
☒	☐	For null hypothesis testing, the test statistic (e.g. F, t, r) with confidence intervals, effect sizes, degrees of freedom and P value noted<br>*Give P values as exact values whenever suitable.*
☒	☐	For Bayesian analysis, information on the choice of priors and Markov chain Monte Carlo settings
☒	☐	For hierarchical and complex designs, identification of the appropriate level for tests and full reporting of outcomes
☒	☐	Estimates of effect sizes (e.g. Cohen's d, Pearson's r), indicating how they were calculated

Our web collection on statistics for biologists contains articles on many of the points above.

Software and code

Policy information about availability of computer code

Data collection	Crystallography data collection was conducted remotely at NE-CAT beam line 24-ID-E at the Advanced Photon Source. The beam line/data collection were controlled using their standard in-house program.
Data analysis	Crystallography data analysis was conducted using the HKL2000v717, PHENIX v1.12, COOT 0.91, and PyMol 2.0.6 software. Ubiquitin thioester activity data analysis was conducted using ImageJ 1.53 and GraphPad Prism 7.0a. Thermal Shift data was analyzed by GraphPad Prism 7.0a.

For manuscripts utilizing custom algorithms or software that are central to the research but not yet described in published literature, software must be made available to editors and reviewers. We strongly encourage code deposition in a community repository (e.g. GitHub). See the Nature Portfolio guidelines for submitting code & software for further information.

Data

Policy information about availability of data

All manuscripts must include a data availability statement. This statement should provide the following information, where applicable:

- Accession codes, unique identifiers, or web links for publicly available datasets
- A description of any restrictions on data availability
- For clinical datasets or third party data, please ensure that the statement adheres to our policy

Atomic coordinates and structure factors are deposited in the RCSB with accession code 7SOL. Coordinates and structure factors for protein structures discussed or used in analysis are accessible at the RCSB. The structural data used from RCSB are listed below: PDB: 6DC6 (https://www.wwpdb.org/pdb?id=pdb_00006DC6), PDB: 4II2 (https://www.wwpdb.org/pdb?id=pdb_00004II2), PDB: 6GF2 (https://www.wwpdb.org/pdb?id=pdb_00006GF2).
The source data underlying Fig. 4a-e, Fig 5d-e, Supplementary Fig. 4 and Supplementary Fig. 5 are provided as a Source Data file.

Field-specific reporting

Please select the one below that is the best fit for your research. If you are not sure, read the appropriate sections before making your selection.

- [x] Life sciences
- [ ] Behavioural & social sciences
- [ ] Ecological, evolutionary & environmental sciences

For a reference copy of the document with all sections, see nature.com/documents/nr-reporting-summary-flat.pdf

Life sciences study design

All studies must disclose on these points even when the disclosure is negative.

Sample size	No sample size calculation was performed. All biochemical experiments (activity assays, thermal shift assay) reported in Figures 4 and 5 were conducted in triplicate since this sample size has been broadly used and accepted by the scientific community for such experiments. Data are represented as mean ± SD of three independent experiments.
Data exclusions	No data were excluded from the analyses.
Replication	n=3 (technical replicates) for all in vitro reactions in Figures 4 and 5. All attempts at replication were successful and were included.
Randomization	This study does not involve animals and/or human research participants and we did not use experimental grouping in these studies, so no randomization was necessary.
Blinding	Not relevant for the study, samples not blinded. No bias could be introduced by the subject or the tester in the experiments performed.

Reporting for specific materials, systems and methods

We require information from authors about some types of materials, experimental systems and methods used in many studies. Here, indicate whether each material, system or method listed is relevant to your study. If you are not sure if a list item applies to your research, read the appropriate section before selecting a response.

Materials & experimental systems

n/a	Involved in the study	
x		Antibodies
	x	Eukaryotic cell lines
x		Palaeontology and archaeology
x		Animals and other organisms
x		Human research participants
x		Clinical data
x		Dual use research of concern

Methods

n/a	Involved in the study	
x		ChIP-seq
x		Flow cytometry
x		MRI-based neuroimaging

Eukaryotic cell lines

Policy information about cell lines

Cell line source(s)	High Five Cells (BTI-TN-5B1-4; Trichoplusia ni) were purchased from Thermo Fisher Scientific.
Authentication	High Five Cells were directly purchased from the vendor, where they were validated. This cell line was not authenticated by the authors. SDS-PAGE results, mass spectrometry, and X-ray crystallography studies confirmed the identity of the expected recombinant proteins expressed in the High Five Cells.
Mycoplasma contamination	High Five Cells were certified as testing negative for Mycoplasma by the vendor. The cells used for recombinant expression of proteins used in our biochemical and structural studies exhibited normal growth pattern and were cultured in a dedicated incubator free from potential contaminating primary or secondary cultures.
Commonly misidentified lines (See ICLAC register)	N/A.
